# Double-level torsional osteotomy a treatment for the ‘inwardly pointing knee’ syndrome

**DOI:** 10.1007/s00402-022-04446-w

**Published:** 2022-05-12

**Authors:** Jens Liße, Mario Perl, Jörg Dickschas

**Affiliations:** 1 Klinik Für Orthopädie Und Unfallchirurgie Klinikum, Buger Straße 80, 96049 Bamberg, Germany; 2 Direktion der Unfallchirurgischen Klinik–Orthopädischen Chirurgie, Krankenhausstraße 12, 2306, 91054 Erlangen, Germany

**Keywords:** Patellofemoral instability, Torsional osteotomy, Anterior knee pain, Inwardly pointing knee, Patellar dislocation, Double level osteotomy

## Abstract

**Introduction:**

A ‘inwardly pointing knee’ syndrome is a combined torsional deformity with increased femoral internal and tibial external torsion. After clinical and radiological verification of the torsional deformity and unsuccessful conservative therapy approach, a combined (double level) torsional osteotomy of femur and tibia might be the appropriate treatment. Here, we present the diagnostic algorithms, treatment, and outcome of combined torsional osteotomies of femur and tibia. The aim of the study is to show that patients treated with the procedure achieve patellofemoral stability and pain relief or reduction.

**Material and methods:**

Twenty torsional osteotomies performed on 18 patients were included. Nine patients had experienced patellar dislocation in 11 joints before. All patients were suffering from anterior knee pain. All patients underwent a clinical and radiographical evaluation, including a torsion angle CT scan. Pre- and post-operatively multiple commonly approved scores (Lysholm Score, Tegner Activity score, Kujala Score, VAS and Japanese Knee Society score) were acquired.

**Results:**

In 18 patients we performed 20 double-level torsional osteotomies. 9 patients suffered from patellar dislocations in 11 knee joints prior to surgery. All patients were suffering from anterior knee pain. Of these 7 patients achieved a stable joint after surgery without further patellar dislocations. All achieved more knee stability and experienced less patellar luxation then before surgery. The mean duration of follow-up was 59 months (range 9–173 months).

The mean VAS was significantly reduced by 3.75 points (SD 2.09, *p* value 0.0002) from 5.50 points (SD 2.73, range 0–9) before surgery to 1.75 points (SD 1.67, range 0–5) after surgery. The Lysholm score increased significantly by mean of 27.6 (SD 17.55, *p* value 0.0001) from mean 62.45 (SD 22.71, range 22–100) before surgery to mean 90.05 (SD 10.18, range 66–100) after surgery. The Kujala Score did improve significantly in average by 25.20 points (SD 13.61, *p* value 0.00012) from mean 62.9 (SD 16.24, range 35–95) to mean 93.2 (SD 9.20, range 66–100). The Tegner activity score did increase significantly by 1.2 points (SD 1.47, *p* value 0.004) in average from mean 2.65 (SD 1.11, range 1–5) to mean 3.85 (SD 1.42, range 1–6). The Japanese knee score did increase significantly by 19.15 in average (SD 11.95, *p* value 0.0001) from mean 74.05 (SD 14.63, range 33–95) to mean 93.05 (SD 10.18, range 68–100).

**Conclusion:**

This is the first publication reporting about simultaneous double-level torsional osteotomies in a comparatively high number of patients. In addition, this is the first publication assessing the patient collective afterwards with objectifying clinical outcome scores. The results show that double-level torsional osteotomy is an effective treatment for patients with patellar dislocation or subluxation associated to torsional deformities of femur and tibia. Furthermore, we introduce a diagnostic algorithm for ‘inwardly pointing knee’ syndrome.

**Level of evidence:**

Level IV.

## Introduction

Anterior knee pain and patellofemoral instability can be caused by several pathologies which lead to patellofemoral maltracking and patellofemoral malalignment: torsional deformities of the lower limb, dysplasia of the trochlear groove, genua valga, increased TTTG distance and patella alta.

In cases caused by a combined increased tibial external torsion and increased femoral internal torsion we are talking about the ‘inwardly pointing knee’ - syndrome. First published and named by Cook et al. [3] in 1990 the clinically appearing inwardly pointing knee was described to be caused by tibial malrotation. Former publications reported on cases of femoral internal or tibial external torsional deformity and a surgical approach to treatment [6, 7]. The publications including isolated tibial or femoral torsional osteotomies showed a significant reduction of anterior knee pain and patellar instability in patients with deformities of the lower or upper limb suffering from these symptoms. But up to now, no study reporting about a representative number of patients treated with double-level torsional osteotomies has been published.

We addressed the following questions in our trial:

- Does double-level torsional osteotomy reduce anterior knee pain in patients with combined femoral and tibial torsional deformity and patellofemoral instability?
- Do patients suffering from patellar dislocation and patellar instability caused by a combined torsional deformity (femoral and tibial) achieve patellar stability when treated with double-level torsional osteotomy?
- Do the patients achieve a better outcome in commonly approved knee scores?

We propose:

- A diagnostic algorithm
- Refined criteria for the ‘inwardly pointing knee’ syndrome

The zero hypothesis was: Double-level torsional osteotomy does not affect anterior knee pain and knee joint stability in patients with combined femoral and tibial torsional deformity.

## Material and methods

### Patients

In a prospective study, 20 double-level torsional osteotomies (femur and tibia) in 18 patients with congenital deformities (16 female, 2 male, age 23.06 years (range 15–47)) performed from 2013 to 2020, were included consecutively. Two patients had surgery on both sides, 16 patients had surgery on only one side. All patients were suffering from anterior knee pain. Nine patients had experienced patellar dislocation.

### Inclusion criteria

1. Pathological torsional values (according to standard values published by Strecker et al. [26] in 1997) of both femur and tibia (raised femoral internal torsion and raised tibial external torsion). We used the standard values established by Waidelich / Strecker with the ‘Ulm Method’ for torsional CT scan measurement:
2. femoral (internal-) torsion angle 20.4° (± 9.0°)
3. tibial (external-) torsion angle – 33.1° (± 8.0°)
4. intraindividual difference upper limb 4.3° (± 2.3°)
5. intraindividual difference lower limb 6.1° (± 4.5°)

They declare a difference of ± 9° femoral torsion and ± 15° tibial torsion as tolerable. In our clinical trial the exact cut-off values were femoral (internal-) torsion angle 33° and tibial (external-) torsion angle – 44°. But these cut-off values must always be considered in the context of clinical symptoms. All patients in our study suffered at least from severe pain with considerable loss of mobility.

2. Anterior knee pain
3. Patellofemoral instability with dislocation or subluxation

For inclusion the first two criteria had to be fulfilled. The third was optional. All patients underwent a long period (6 months minimum) of non-surgical treatment prior to surgery.

### Exclusion criteria

The declared exclusion criteria were age > 50 years, infection, tumor or any other congenital disorder except for patellofemoral malalignment and torsional deformities, as well as valgus deformities with > 10°valgus, open epiphysis, and traumatic injuries of the knee. Furthermore, other pathologies of the patella causing the symptoms such as patella alta or trochlear dysplasia were defined as exclusion criteria. Excluded from the study were also patients with a singular femoral or tibial torsional deformity.

### Clinical investigation

**Fig. 1:**
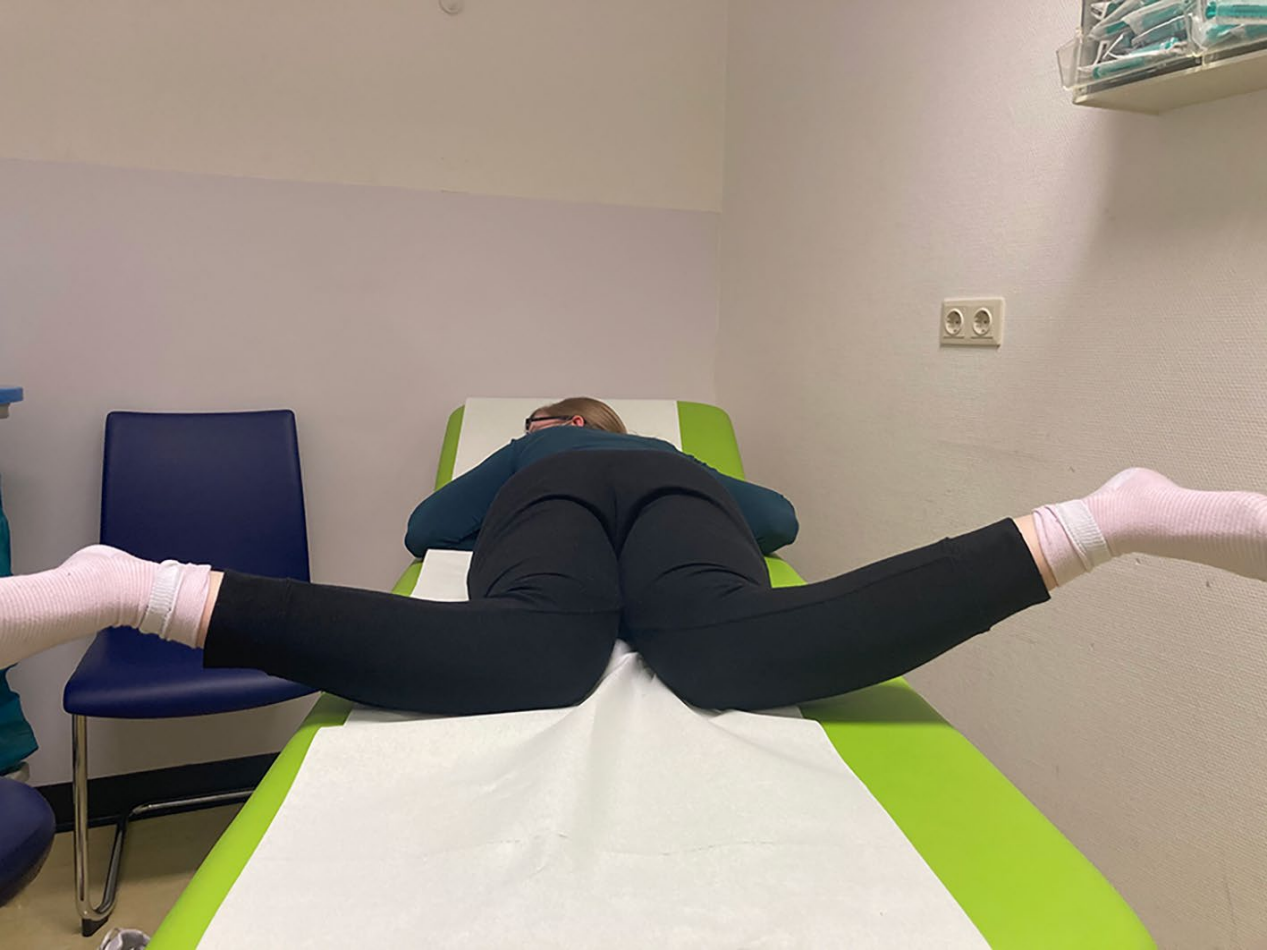
Clinical photographs: Prone position measurement in the clinical examination. The raised femoral internal rotational is obvious

For assessment all patients had a clinical examination focused on the detection of squinting patellae, abnormal torsional profile in prone position, patellofemoral maltracking, and patellar mobility. The range of movement was tested as well as J-sign, apprehension sign, patella tilt, Lachman test, pivot shift test, varus and valgus stress test and anterior and posterior drawer test. Most of the patients (14) were not previously surgically treated before. Four patients had been operated before. Clinical investigation was performed preoperatively and postoperatively after 6 weeks and after full recover (at least 6 months after surgery) (Fig. 1).

### Radiography

**Fig. 2:**
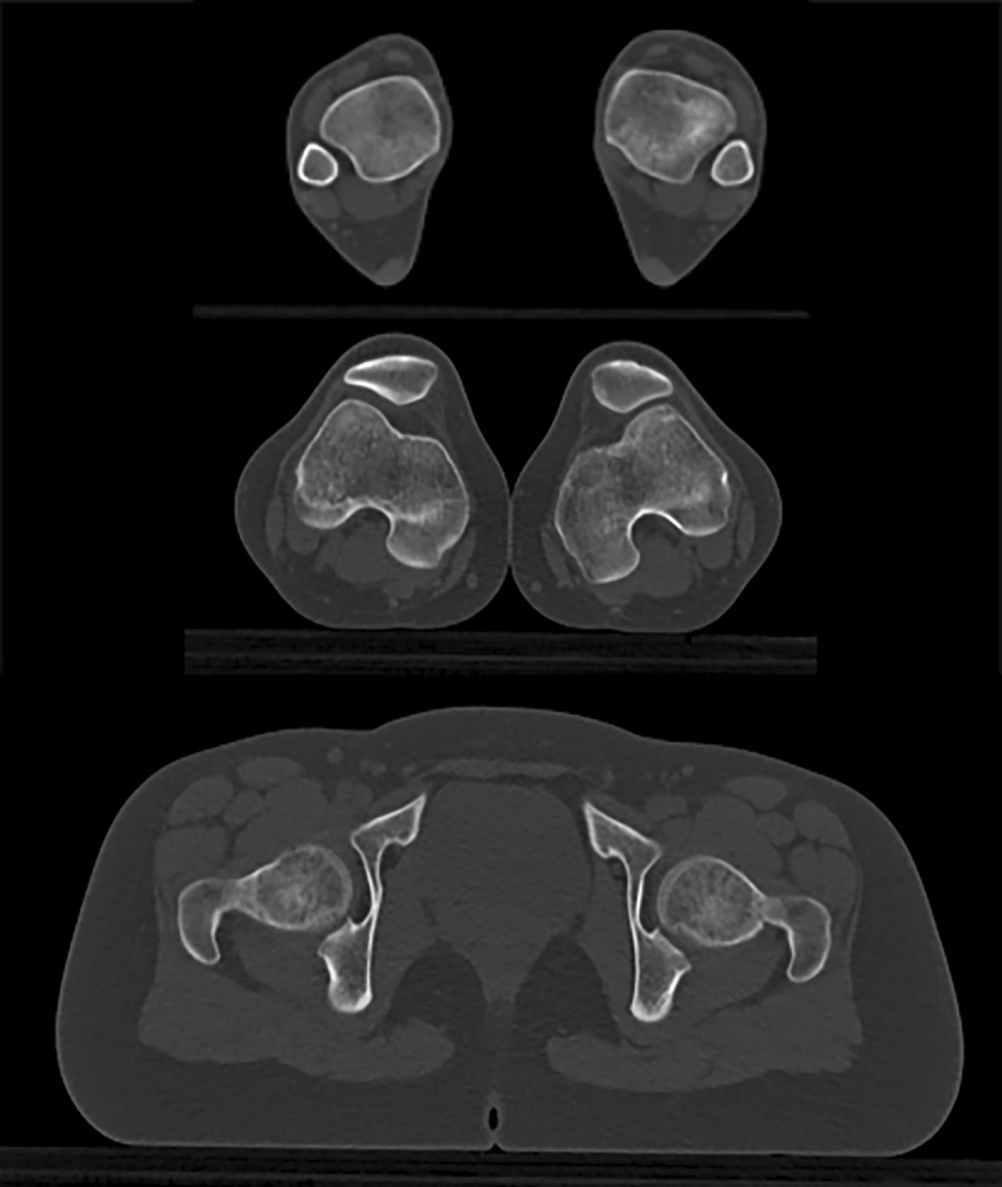
Torsion angle CT scan of a 19 year-old female patient with severe ‘inwardly pointing knee syndrome’. The woman suffered from anterior knee pain and patellar dislocation

Patients who presented with anterior knee pain or patellar instability were admitted for a knee consultation during which a detailed physical examination was performed. In all cases knee x-rays in both planes and full weight bearing long leg views were obtained. In most cases additionally we obtained tangential images of the patella in 30°, 60° and 90° flexion. When an elevated q-angle, a positive J-sign or a raised femoral internal or tibial external torsion was suspected, a torsional CT scan was performed. (Fig. 2).

### Angle of torsion and torsional index

For diagnosis and treatment purposes a torsion-angle computed tomography was performed in all patients prior to surgery. The location of the deformity was identified (femoral and/or tibial) and the magnitude of the torsional deformity quantified using Waidelich’s [12, 29] ‘Ulm method’. The standard values published by Strecker et al. [26] in 1997 were used for this purpose. The torsional index introduced by Dickschas et al. [6] was calculated for every patient and every affected joint. Therefor torsion measures valued by Strecker et al. [26] were used as a standard.

### Surgery

All patients were treated with a combined femoral and tibial torsional osteotomy. In all patients, we performed an arthroscopy prior to osteotomy to evaluate the state of the cartilage and the patellofemoral balance as well as treat other intraarticular pathologies if necessary. All arthroscopic findings were imaged and recorded. In 2 cases a plica resection had been done arthroscopically prior to osteotomy.

For tibial osteotomy we accessed the tibial head in a lateral approach. The anterior tibial fascia was incised, and the proximal portion of the anterior tibial muscle carefully dissected. The lateral tibial head was reached and Gerdy’s tubercle identified. In one case a lateral retinaculum release and in one case a lateral retinaculum plasty to the proximal edge of the patella had been done prior to osteotomy. The osteosynthesis plate (5-hole DCP) was then bent to right shape.

**Fig. 3:**
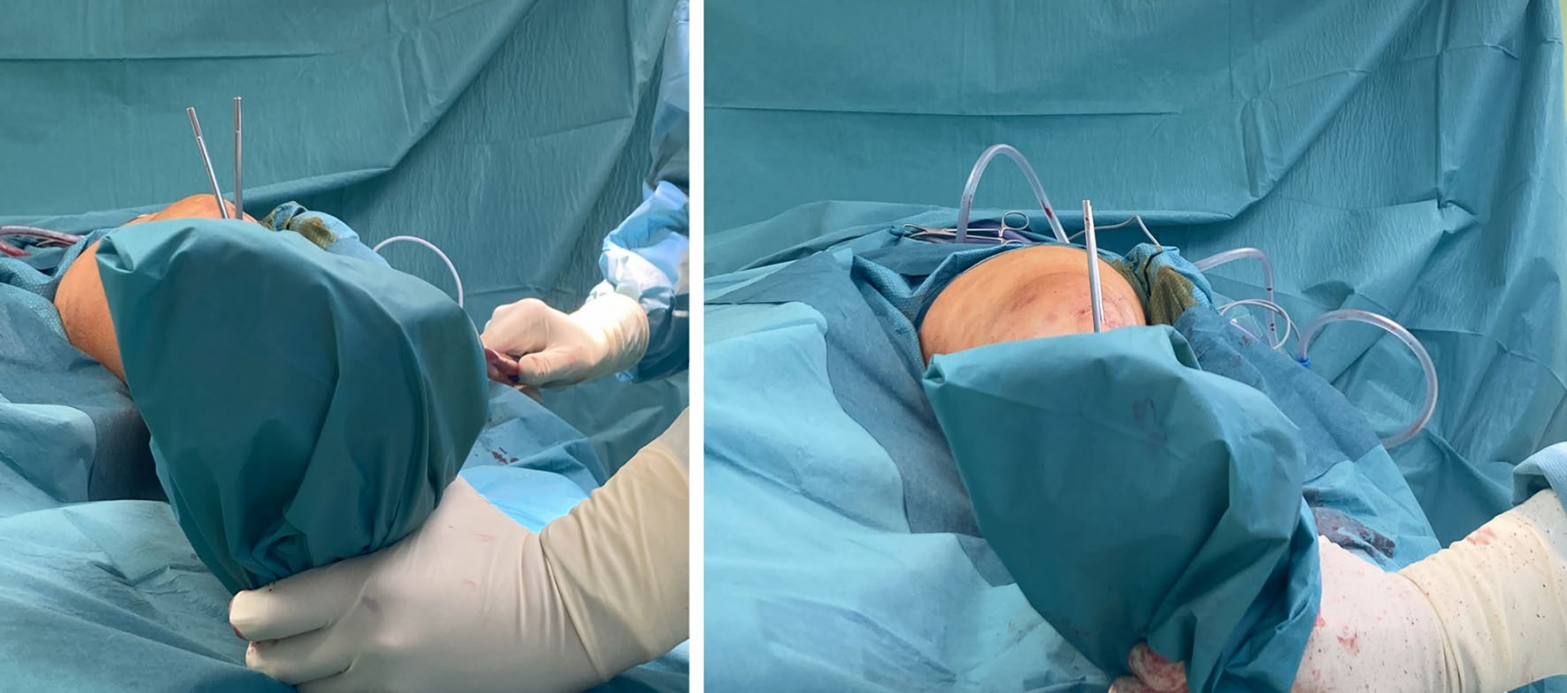
Axial view: Intraoperative pictures performing a tibial internal osteotomy via lateral approach. The two positioned Schanz screws–one proximal and one distal to the osteotomy level—are used as torsional indicators to execute the torsional correction. After correction both Schanz screws are congruent on the axial view

Two 5 mm Schanz screws were placed for torsional measurement and intraoperative torsional control. One screw was placed distal and one proximal to the planned osteotomy plane to indicate the torsional angle. Both were placed without interference to the subsequent base of the osteosynthesis plate. The two screws were set in the exact torsional angle of correction which should be achieved (Fig. 3).

**Fig. 4:**
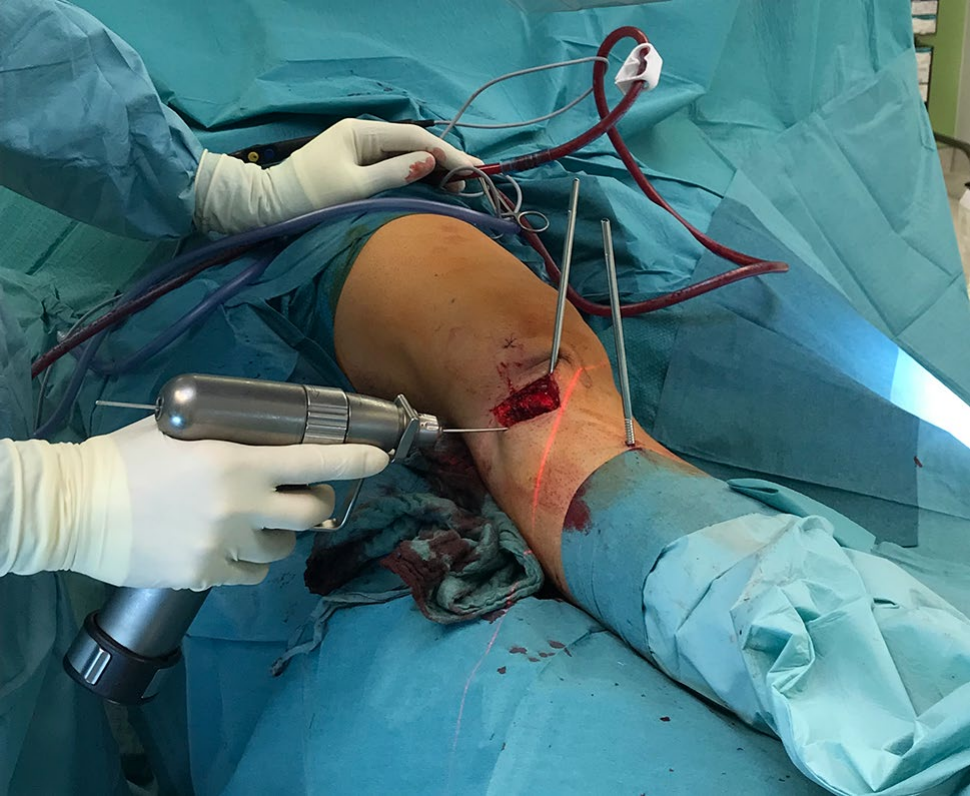
Intraoperative picture performing a double-level osteotomy. Here the tibial internal osteotomy is done via lateral approach. The two Schanz screws have been positioned–one proximal and one distal to the osteotomy level. They are used as torsional indicators to execute the torsional correction. In this step the K-wire is placed to mark the level for the osteotomy

**Fig. 5:**
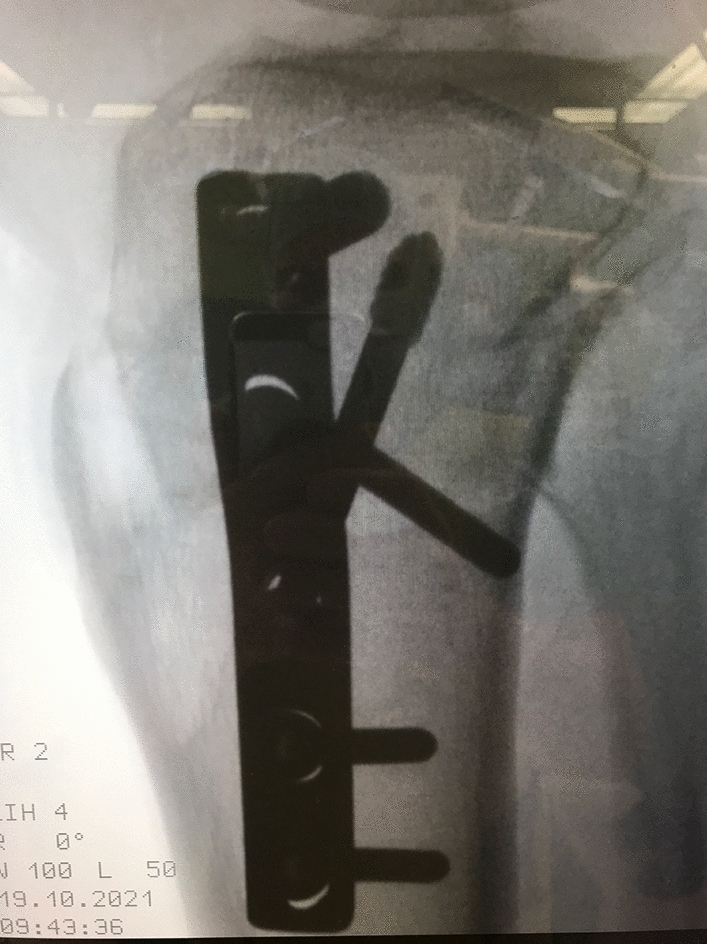
Intraoperative x-ray lateral view after the tibial internal torsional osteotomy as the first step of a double-level torsional osteotomy. The osteotomy was performed and osteosynthesis was done with a 5-hole DCP plate tibial. In the lateral view the infratuberositary level of the osteotomy leading to a supratuberositary level at the ventral aspect can be seen

This was measured with a sterile angle gauge reed in axial view of the leg. A *K*-wire was used marking the level of osteotomy. It was placed exactly 90° to the mechanical tibial axis (Fig. 4). The osteotomy was done with an oscillating saw while protecting soft tissue in the ventral aspect (patellar tendon) and the dorsal aspect with the Hohmann retractor. Following was a second osteotomy at a 110° angle to the main osteotomy, about 1 cm dorsal to the tibial tubercle. (Fig. 5) To avoid arterial or neural damage special care was taken while working in the dorsal aspect.

Next the two Schanz screws were used for measuring the angle of correction. The distal tibia was carefully rotated internally until a parallel stand (axial view of the leg) of the two Schanz screws was achieved, indicating the planned torsional angle.

The procedure at the tibia was finished by fixation with the priorly bent osteosynthesis plate.

To avoid damage of the peroneal nerve maximum internal correction should not exceed 15°. We monitored all patients 48 h postoperatively for signs of a compartment syndrome.

For femoral osteotomy we accessed the distal femur medially performing an incision about 10 cm. The fascia of the femur was incised and the medial vastus of the quadriceps femoris muscle was dissected. The medial aspect of the femoral condyle was reached and the subsequent base for the osteosynthesis plate prepared.

Analogous to the procedure at the tibia two Schanz screws—one distal and one proximal to the level of the planned osteotomy—were set for torsional measurement and intraoperative torsional control. The osteotomy was performed supracondylar [22].

**Fig. 6:**
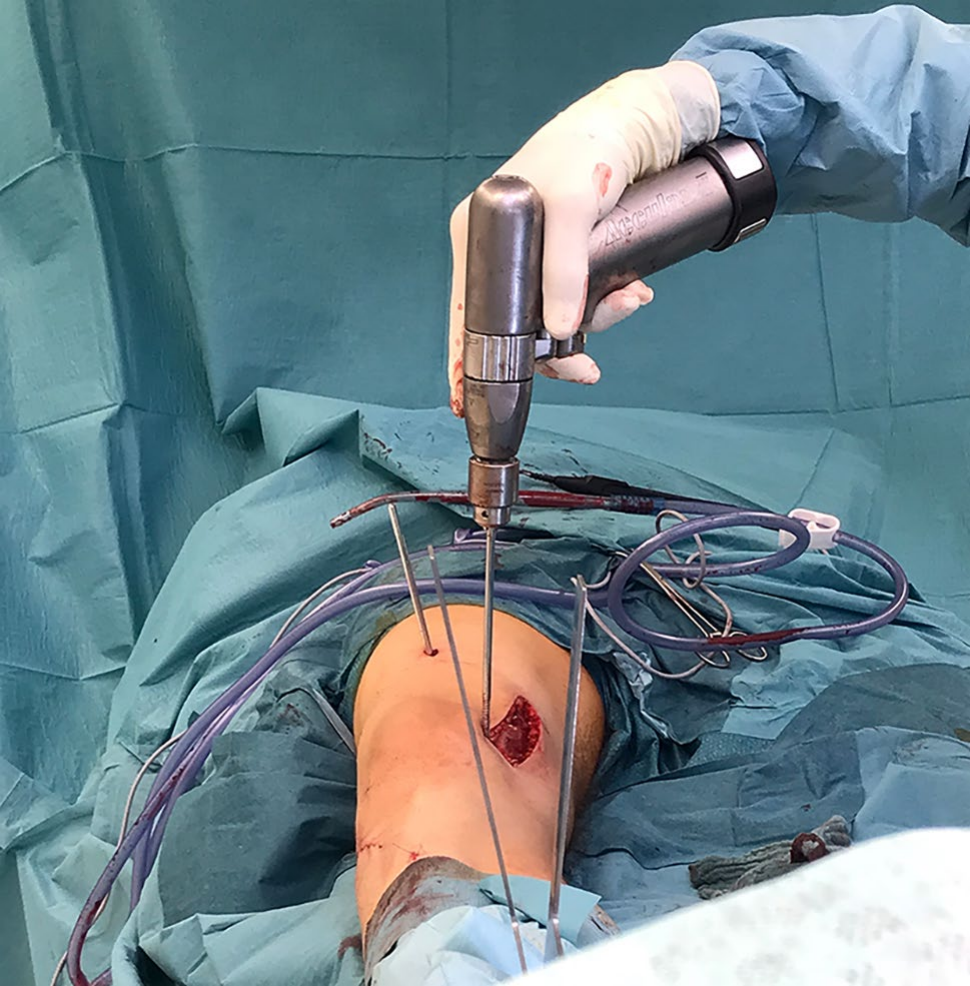
Axial view: Intraoperative picture performing the femoral external torsional osteotomy as second step of a double-level torsional osteotomy. Positioning of the two Schanz screws for the torsional measurement. The two screws were set in the exact torsional angle of correction which should be achieved. This was measured with a sterile angle gauge reed in axial view of the leg

**Fig. 7:**
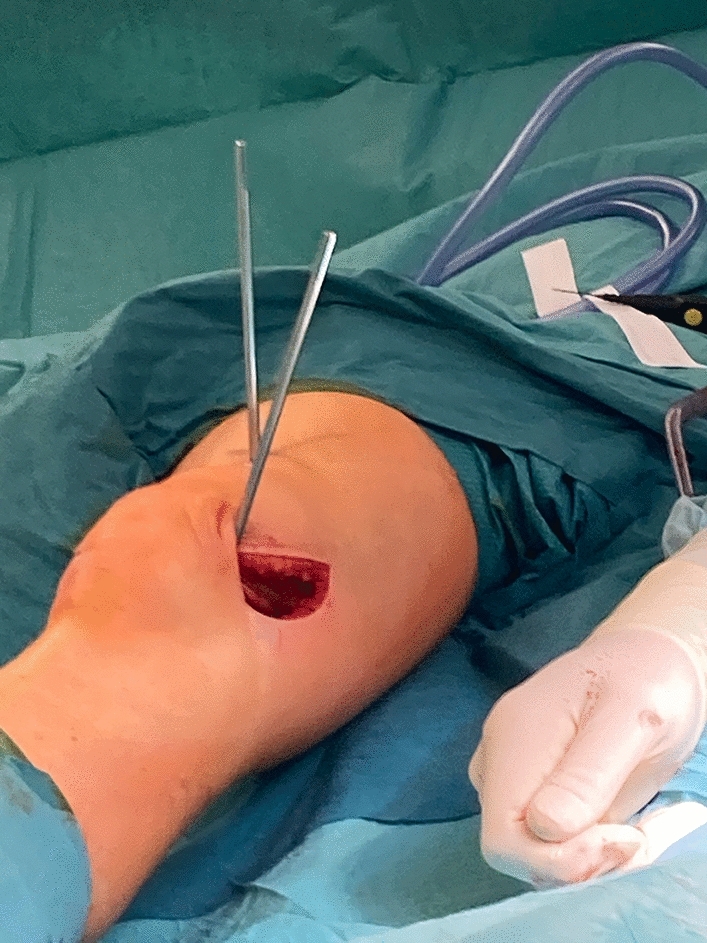
Intraoperative torsional measurement with two Schanz screws as torsional indicators performing a femoral external rotational osteotomy, medial approach

**Fig. 8:**
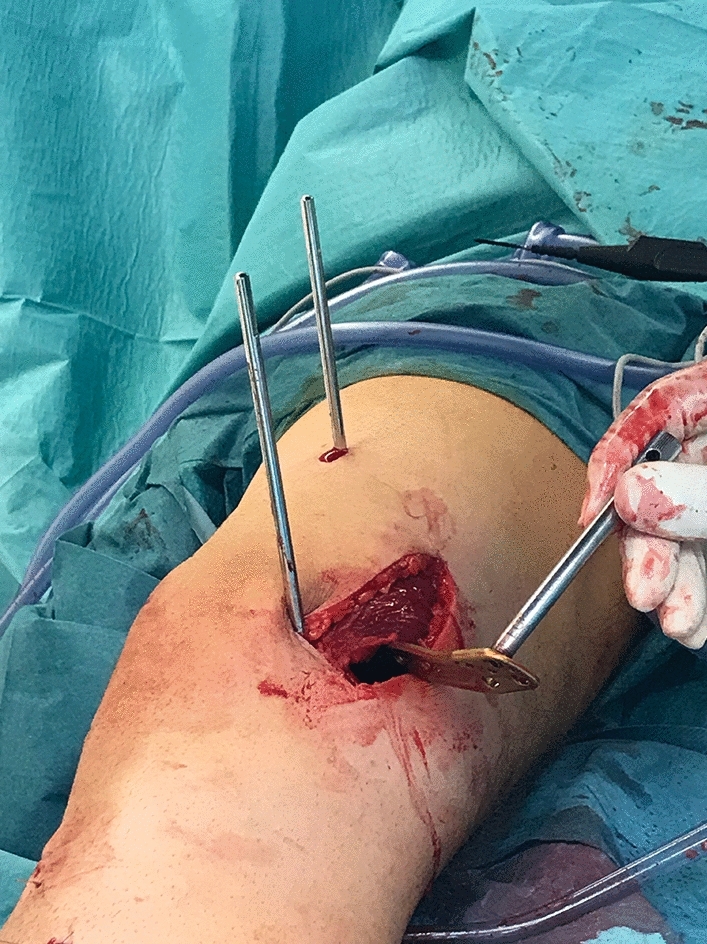
Intraoperative picture performing the femoral external torsional osteotomy as second step of a double-level torsional osteotomy. The supracondylar osteotomy has been performed and as the two Schanz screws standing in parallel position indicate the torsional correction has been achieved. The MDF TomoFix plate is being insert

Both were placed without interference to the subsequent base of the osteosynthesis plate. The two screws were set in the exact torsional angle of correction which should be achieved. This was measured with a sterile angle gauge reed in axial view of the leg (Figs. 6, 7). A *K*-wire was used marking the level of osteotomy. The implants used for osteosynthesis were MDF TomoFix plates femoral (Fig. 8).

### Additional surgical procedures

In two cases we addressed the additional minor (< 10°) varus deformity with valgisation osteotomies.

In one case a lateral retinaculum plasty and in one case a lateral release had been done additionally. The indication for both was an intraoperatively in the tissue laterally strongly fixed patella.

As mentioned before in three cases of severe knee instability and receding patellar dislocations the double-level osteotomy reduced the frequency of occurrence decisively but did not provide a stable joint. Two of the three patients achieved a stable joint after an additional Medial Patellofemoral Ligament reconstruction. The third patient rejected any further surgery.

In two cases we addressed a diagnosed plica syndrome (whilst the previous arthroscopy) with a plica resection. The indication was a clamping plica at arthroscopy.

### Post-operative treatment

**Fig. 9:**
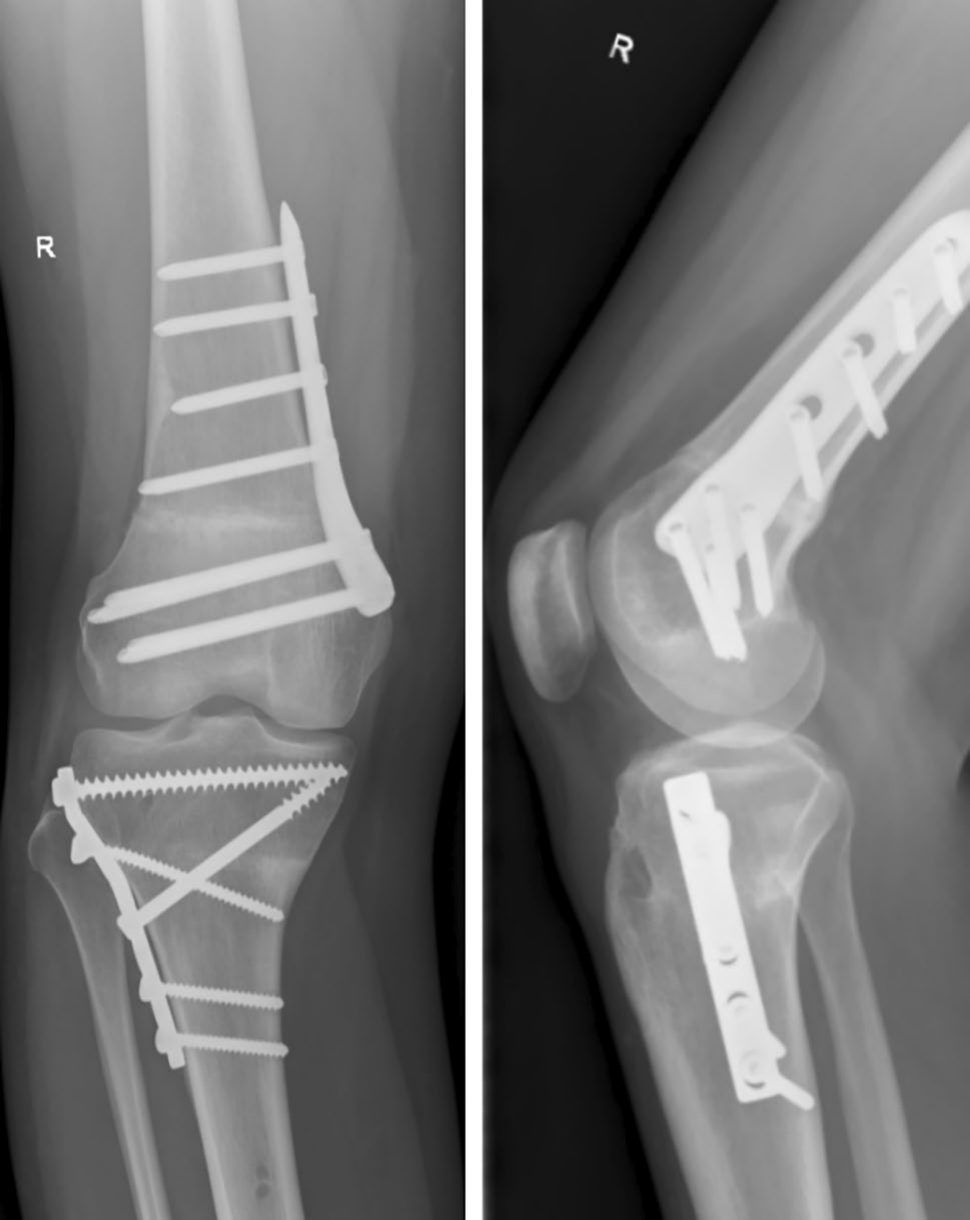
Radiography of the knee in two planes of a 21 year-old woman with severe ‘inwardly pointing knee syndrome’ six months after double-level (12°-femoral external and 12°-tibial internal rotational) osteotomy and osteosynthesis (with 5-hole DCP plate tibial and MDF TomoFix femoral), bony consolidation

**Fig. 10:**
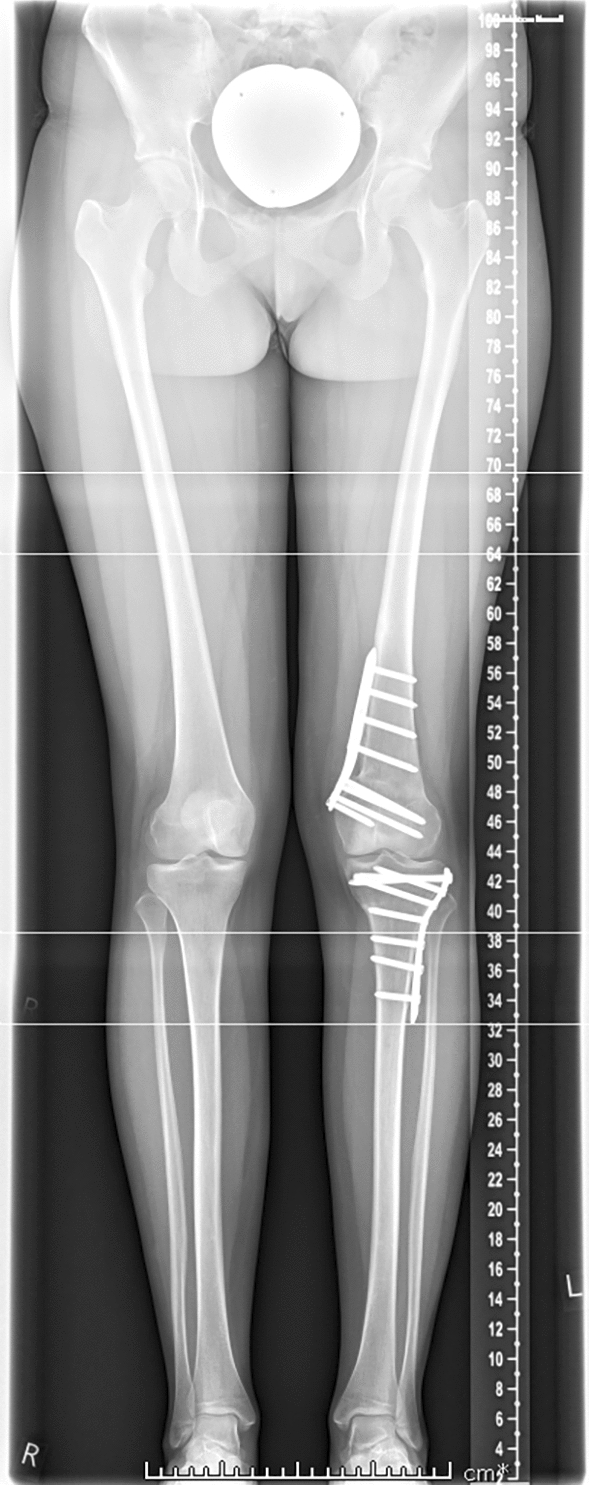
Post-operative x-rays long leg view of a 17 year-old female 10 months after double level osteotomy (femoral external and tibial internal rotational osteotomy) and osteosynthesis, bony consolidation

Mobilization of the knee started immediately the first day after surgery with continuous passive motion (CPM) training and physiotherapy. Patients were mobilized with forearm crutches, with 20 kg of partial weight-bearing on the operated leg. X-rays were obtained after 6 weeks, and loading was permitted at this time. Post-operatively a clinical examination as well as radiography was performed. The radiographic imaging included knee in two planes and full weight-bearing whole leg view. According to the young age of the patients and to keep radiation exposure small no post-operative torsional angle CT scan was performed (Figs. 9, 10).

### Follow-up examination

The mean duration of follow-up was 59.05 months (range 9–173 months). The follow-up examination consisted of an inquiry of the below-mentioned scores and a clinical examination. It was like the initial examination. The results of the follow-up examination were recorded by the first author. The follow-up examination was done 6 weeks and 6 months after surgery.

### Subjective/objective scores

The following scores were used for assessment prior and after treatment:

- Lysholm score [17]
- Japanese Knee Society score [20]
- Kujala score [4]
- Tegner activity score [27]
- Visual analog scale (VAS)

### Statistical analysis

**Fig. 11:**
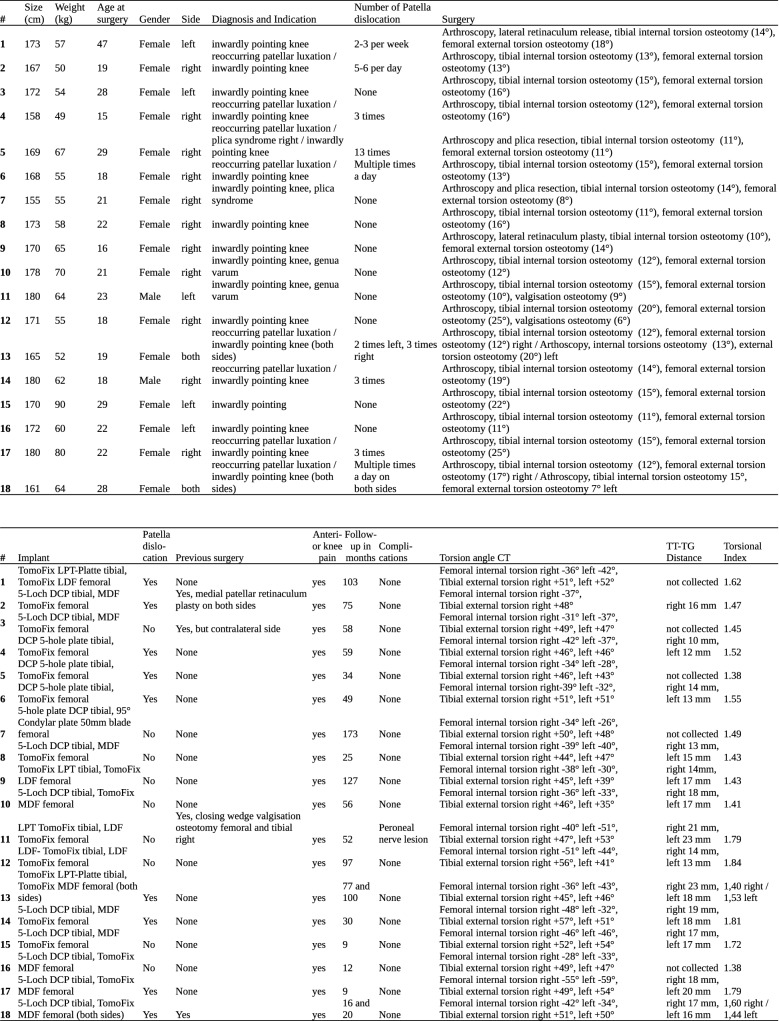

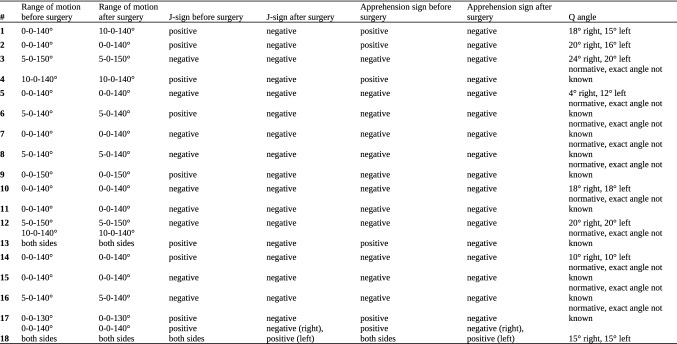
Anonymized list of all included patients with detailed information on age, gender, weight, height, operated side, surgery, implants, previous surgery, additional procedures, results of clinical examination, diagnoses and indication, symptoms (including number of patellar dislocations), follow-up period and findings in imaging

Statistical analysis was performed with Microsoft Excel and Python modules NumPy and SkiPy. To determine the magnitude of difference of the matched groups and prove probability of the wrong rejection of the zero hypothesis Wilcoxon’s signed rank test was used. The probability of wrong rejection of the zero hypothesis (no group differences) was set to a significance level of *p* < 0.05 (Fig. 11).

## Results

The mean duration of follow-up was 59.05 months (range 9–173 months). Analysis of the torsional CT scans gave a mean femoral torsion of – 40.85° (SD 6.25°, range – 33° to – 55°) and a mean tibial torsion of 49.15° (SD 3.71°, range 44°–57°) evaluated before surgery. The mean torsional index in the affected legs was 1.55 points (range, 1.38–1.84 points).

### Patella dislocations

Of the 18 treated patients 9 had patellar dislocations in 11 knee joints (two patients on both sides) prior to surgery. Of these patients 6 achieved a stable joint after surgery without further patella dislocations. In two cases an additional Medial Patellofemoral Ligament reconstruction was necessary to achieve stability. In the third case the patient preferred to keep status quo before surgery. Nevertheless, the patient achieved more knee stability and experienced less patellar dislocations (from 2–3 dislocations/day before to 1–2 dislocations/year after) then before surgery.

### Results of examination/clinical assessment

The mean VAS was significantly reduced by 3.75 points (SD 2.09, *p* value 0.0002) from 5.50 points (SD 2.73, range 0–9) before surgery to 1.75 points (SD 1.67, range 0–5) after surgery. The Lysholm score increased significantly by mean of 27.6 (SD 17.55, *p* value 0.0001) from mean 62.45 (SD 22.71, range 22–100) before surgery to mean 90.05 (SD 10.18, range 66–100) after surgery. The Kujala Score did improve significantly in average by 25.20 points (SD 13.61, *p* value 0.00012) from mean 62.9 (SD 16.24, range 35–95) to mean 93.2 (SD 9.20, range 66–100). The Tegner activity score did increase significantly by 1.2 points (SD 1.47, *p* value 0.004) in average from mean 2.65 (SD 1.11, range 1–5) to mean 3.85 (SD 1.42, range 1–6). The Japanese knee score did increase significantly by 19.15 in average (SD 11.95, *p* value 0.0001) from mean 74.05 (SD 14.63, range 33–95) to mean 93.05 (SD 10.18, range 68–100).

**Fig. 12:**
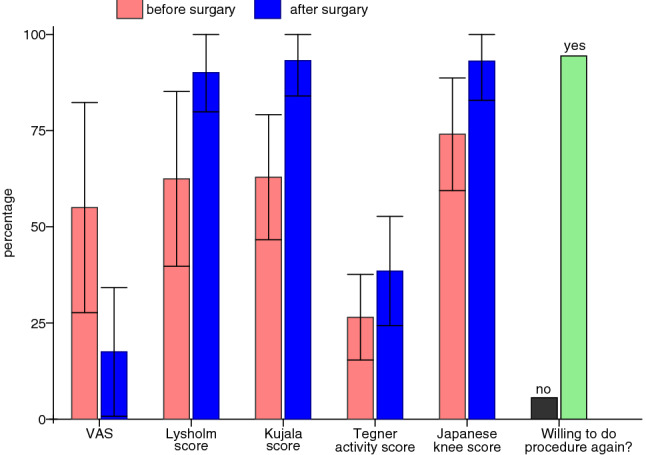
The table shows the outcome (in percentage) for the 18 patients in the clinical scores: VAS, Lysholm, Kujala, Tegner activity score, Japanese knee score and if they are willing to do the procedure again. For the scores the standard deviation is shown

Of 18 patients 94.4% or 17 patients were willing to undergo the procedure again. The only patient who would not undergo the procedure again was one patient with the complication of a postoperative peroneal nerve affection (Fig. 12).

Of 21 patients having achieved double-level torsional osteotomies in our department 3 patients were lost to follow-up. This led to inclusion of 85.7% of the patients in the study.

### Complications

In one case a peroneal nerve lesion occurred. Early after surgery there was a strong peroneal nerve palsy which recovered nearly completely. Until the time of follow-up (52 months) the patient had a persisting minor foot lifting weakness. No non-unions occurred in the treated patients [9].

## Discussion

The results of the study show that double-level torsional osteotomy is an effective treatment option for patients with ‘inwardly pointing knee' syndrome. Furthermore, the results show that all patients who suffered of patellar dislocation achieved more knee stability and less patellar dislocations. All patients improved in the clinical outcome scores which means they achieved more mobility and suffered less pain than before surgery.

The ‘inwardly pointing knee’, ‘torsional malalignment (TMS)’ or ‘miserable malalignment’ syndrome is a rare and unique but important reason for anterior knee pain and patella dislocation or subluxation in mainly young women with congenital torsional leg deformity. Only severe cases—refractory to conservative treatment—require surgery. Nevertheless, in patients with anterior knee pain or patella dislocations a variety of potential reasons may be responsible. Therefor a good diagnostical algorithm giving the right indication and finding a suitable surgical technique to address the pathology is required. Torsional deformities are still an underestimated reason for the above-mentioned symptoms.

The first reports of torsional osteotomies go back to Lowman 1919 [16], O’Donoghue 1940 [19] and Levine et al. [15]. In 1957 Somerville described 8 patients (aged 5–21 years) successfully treated with double-level torsional osteotomies. In his opinion the treatment addressed a ‘defective gait rather than a deformity demonstrable radiologically’ [23]. The technological improvement and implementation of CT-scans with torsional measurements [12] changed this point of view and radiologically illustrates the problem: A torsional deformity. Furthermore, today an operative treatment of torsional deformities is preferably done after the end of bone growth. In 1989 Staheli [24] stated that’these deformities are relatively common in infancy and childhood, generally resolve spontaneously with growth, and rarely persist into adult life.’ Recent publications also show that torsional deformities of both the femur and tibia can be successfully treated with torsional osteotomies [13, 28]. Frosch et al. point out that torsional deformities can be the reason for patellofemoral maltracking [10].

James [11] introduced the term ‘miserable malalignment’ in 1979 to describe patients with excessive femoral anteversion, increased Q-angle, and excessive outward tibial torsion. Meaning the same 1990 Cooke et al. [3] described the ‘inwardly pointing knee’ syndrome and an approach to treatment. They report about surgery in seven cases (9 knees) with derotation-valgus (Maquet) osteotomy of the tibia and lateral release. But only five knees had a follow-up, and no clinical scores were evaluated. They already proclaimed that ‘it is recommended that patients with inwardly pointing knees and anterior knee pain be routinely screened for torsional abnormalities’ [3].

There a several studies reporting about torsional osteotomies as a treatment for an isolated tibial or femoral torsional deformity. Benett et al. [1] performed 32 rotational osteotomies of the distal tibia in 19 patients with a high success rate. Meister et al. [18] also report about 7 cases of tibial osteotomy in patients with severe torsional deformity of the tibia with good results in an average of 10 years follow-up. Dickschas et al. [7] performed 30 distal femoral torsional osteotomies in 25 patients and 45 tibial torsional osteotomies [8] in 42 patients. The leading symptoms were anterior knee pain and patellofemoral instability. They report about good to excellent results evaluated with Tegner’s, Lysholm’s, and Japanese knee society score. Dickschas et al. [6] also evaluated 32 torsional osteotomies with 11 isolated femoral and 19 isolated tibial osteotomies. They assessed their patients with clinical scores and showed a significant improvement, less pain and achieved knee stability. Several surgical approaches to torsional osteotomy are known and established [21].

Until now there exist only a few publications on double-level torsional osteotomies. None of them evaluated their patients with approved clinical scores. Delgado et al. [5] applied combined (femoral and tibial) torsional osteotomies for their group of nine patients with ‘miserable malalignment’ and claim good results with surgical treatment.

Leonardi et al. [14] reported about 12 surgeries in 9 patients with torsional deformities. But only in three cases a double-level torsional correction was performed. No clinical scores for the objectification of success were evaluated. Nevertheless, they stated ‘in cases of significant deformity, internally rotating the tibia alone is not sufficient.’ According to Staheli [25] knee pain is most likely to occur with the combination of femoral internal torsion if tibial external torsion is also present. He further stated that double-level osteotomies are effective in relieving the knee pain in these patients, although operative treatment is only rarely indicated.

The highest number of patients with double level torsional osteotomies so far were evaluated by Bruce et al. [2]. They report on 14 patients with 27 involved limbs with ‘miserable malalignment’ syndrome suffering from patellofemoral pain. All patients were treated by rotational osteotomy of the femur and tibia. They performed 20 distal tibia osteotomies and 7 proximal tibia osteotomies. The femur osteotomy was done supracondylar in 13 cases and intertrochanteric in 6 cases. In their patients only few complications occurred (one femoral shaft-fracture, one loss of tibial fixation and one fibular non-union) but none of the complications were persistent. They report about good results but give no evidence of their results with clinical scores.

Yet this is the first publication reporting about simultaneous double-level torsional osteotomies in a comparatively high number of patients. In addition, this is the first publication assessing the patient collective afterwards with objectifying clinical outcome scores.

All 18 patients fitted the definition of an ‘inwardly pointing knee’ according to Cooke et al. [3] established in 1990. To diagnose an ‘inwardly pointing knee’ syndrome three of five criteria must be fulfilled:

- 1. Inwardly turned knee joints in standing position.
- 2. Chronic retropatellar pain.
- 3. Patellar dislocation or subluxation.
- 4. Retropatellar induced instability (giving way).
- 5. Genua vara et recurvata.

Yet the last of the five criteria ‘genua vara et recurvata’ should be questioned. In only 2 of the 18 patients the fifth criteria matched.

**Fig. 13:**
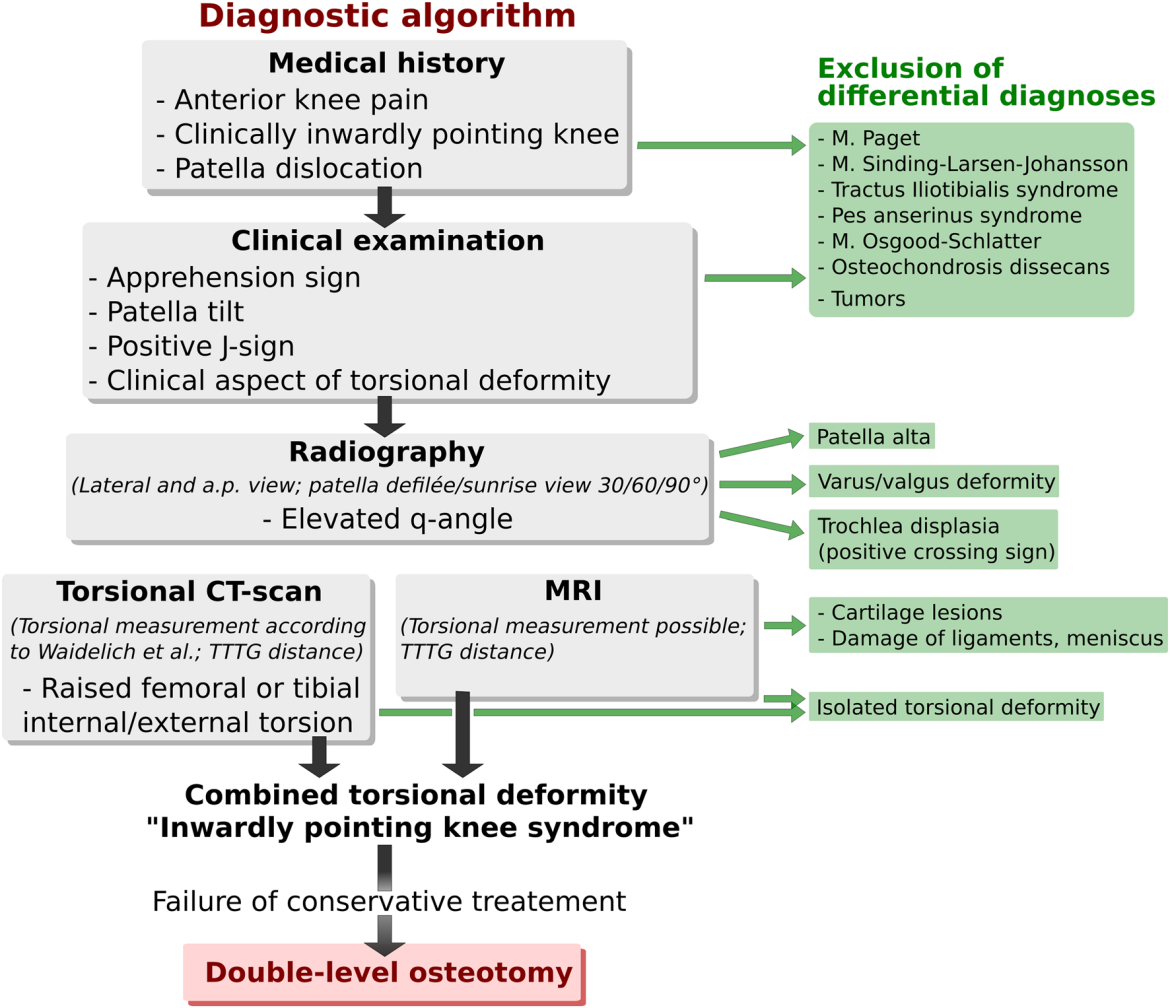
Diagnostic algorithm for ‘inwardly pointing knee’ syndrome

We propose a diagnostic algorithm to give the indication for double-level torsional osteotomy (Fig. 13). The algorithm shows the path that can lead to the indication for double-level osteotomy. The algorithm represents the approach in department and has not been validated. The patient history includes anterior knee pain (especially when climbing stairs) in all cases and patellar dislocations in about half of the cases. Patients with dislocations usually have a positive J-sign or apprehension sign on clinical examination. Visually, the inwardly pointing knee is often already noticeable. Sometimes conventional radiography shows an increased Q angle. In the x-ray sunrise view, subluxation or dislocation positions of the patella can be detected. However, the conventional radiographs are mainly used to exclude differential diagnoses. The final diagnosis can only be made with a torsion angle CT or MRI scan. After a failed conservative therapy attempt of at least 6 months, the indication for a double-level osteotomy can be given.

The torsional index established by Dickschas et al. [6] has been calculated for all patients. The mean torsional index in the affected legs was 1.55 points (range, 1.38–1.84 points), this proofs that the established physiological range of 0.8–1.2 has been exceeded in all cases and in return that the torsional index is a good parameter for the severity of a torsional deformity. Nevertheless, there was no significant correlation found in the spearman’s correlation rank test (spearman correlation coefficient 0.27, two-sided *p *value 0.24) between VAS scores and torsional index.

Of course, in comparison to other surgical approaches the number of patients being evaluated was still small. Due to the rarity of the severe ‘inwardly pointing knee’ syndrome and the cases occurring in one orthopedic department alone for a stronger statistical representation multicenter or meta-analysis studies should be a considerable future option. For a higher level of evidence, a comparison with a control group would be considerable. But the inclusion of a control group was not possible because it would have been ethically incorrect to offer no treatment to patients with such severe symptoms.

According to the young age of the patients and to keep radiation exposure small no post-operative torsional angle CT scan was performed. It is arguable to establish MRI-scans or low dose CT-scans after surgery for post-operative torsional measurement and therefor more objectivation.

Four of the patients had surgical treatment before which could be a bias influencing the evidence. Additional procedures like varisation or valgisation osteotomy in 6 cases could also be a source of bias.

## Conclusion

The results show that double level torsional osteotomy is an effective treatment option for patients with patellar dislocation or subluxation associated to torsional deformities of femur and tibia. The patients achieved joint stability through the procedure. In the only case with persisting instability the occurrence of patellar dislocation or subluxation was significantly reduced.

Furthermore, the results indicate that anterior knee pain associated to combined torsional deformity of femur and tibia can significantly be reduced by a double-level osteotomy. The level of activity patients achieve through the treatment enhances significantly as well. With correct indication and an appropriate diagnostic algorithm as well as exclusion of other pathologies our results show that double-level torsional osteotomy is an appropriate and reliable surgical therapy to achieve joint stability and reduce pain.

We propose a diagnostic algorithm to give the indication for double-level torsional osteotomy.

We propose a refinement of Cooke’s definition of the criteria fitting the ‘inwardly pointing knee’ syndrome and summarize the terminology:

The ‘inwardly pointing knee’ syndrome, ‘torsional malalignment’ syndrome (TMS) or ‘miserable malalignment’ syndrome consists of:

1. Inwardly turned knee joints in standing position (clinical examination).
2. Chronic retropatellar or reported anterior knee pain refractory to conservative treatment (medical history).
3. Patellar dislocation or subluxation (medical history, radiography).
4. Retropatellar induced instability, so called ‘giving way’ (medical history, clinical examination).
5. An increased femoral internal and tibial external torsion (torsional CT- or MRI scans).
